# “Letter to the Editor: Assessing Knowledge and Practices Towards Routine Medical Checkups Among Medical Students of Karachi: A Cross‐Sectional Study”

**DOI:** 10.1002/hsr2.72784

**Published:** 2026-07-19

**Authors:** Muhammad Mehr Ali, Yousra Noor, Amna Khalid

**Affiliations:** ^1^ Northwest School of Medicine Peshawar Pakistan; ^2^ Ayub Medical College Abbottabad Pakistan; ^3^ Federal Medical College Islamabad Pakistan

## Conflicts of Interest

The authors declare no conflicts of interest.

Editor,

We read with great interest the study by Akram et al. (2026), “Assessing Knowledge and Practices Towards Routine Medical Checkups Among Medical Students of Karachi: A Cross‐Sectional Study.” [1] We commend the writers for tackling a current and significant issue: the discrepancy between future physicians' theoretical understanding and their real preventive health practices in a low‐middle‐income nation. It is praiseworthy that four public medical colleges in Karachi were included in the multicentric design and that both the knowledge and practice domains were measured. The fact that the study creates opportunities for further interventional research to enhance preventative health habits throughout Pakistani society is commendable.

But in order to present a fair picture, some flaws should be acknowledged. Therefore, we would like to draw attention to a number of methodological issues that have not been addressed by the authors themselves and that may have a significant impact on how the given findings are interpreted and applied.

First, there was no verification against medical records or institutional health service data in the practice section of the questionnaire (Table 5), which only depended on students recalling when they last had a checkup. This creates recall bias, especially for events that occurred more recently. For example, a student who had a checkup more than a year ago would not recall it correctly, while a student who never had one might mistake a sick visit for a preventive check. Because of this, the already startling 26.8% of those who said they had never had a checkup may be overestimating or underestimating the actual percentage, making it difficult to determine the exact size of the knowledge‐practice gap. Short memory windows or record‐based verification should be used whenever feasible, as Bhandari and Wagner showed that self‐reported recall of health service consumption is prone to considerable mistake, particularly for occurrences beyond 6–12 months [2].

**Table 4:** Comparison of mean values of the knowledge of respondents towards routine medical check‐ups in relation to their personal characteristics (*N* = 246).

	Mean (SD)	Test statistics	*p* value
*Sex (t)*			
Female	3.58 (0.991)	0.227	0.82
Male	3.62 (1.033)		
*Age (f)*			
18–20	3.57 (0.746)	0.078	0.93
21–23	3.58 (1.00)		
24–26	3.64 (1.10)		
**Y** *ear of study (f)*			
Third Year	3.57 (1.046)	0.607	0.55
Fourth Year	3.52 (1.052)		
Fifth Year	3.67 (0.90)		

**Table 5:** Practices and perceptions of undergraduate medical students toward routine medical checkups (*N* = 246).

	Frequency (*n*)	Percentage (%)
*Do you personally schedule regular routine checkups even when you feel healthy?*		
Yes	53	21.4
No	193	78.6
*Have you ever recommended routine checkups to family or friends as a preventive health measure?*		
Yes	198	80.4
No	48	19.6
*When was the last time you had a routine checkup?*		
Less than 6 months ago	52	21.0
6–12 months	53	21.4
1–2 years ago	29	11.6
More than 2 years ago	47	19.2
Never	65	26.8
*Do you feel confident in discussing the benefits of routine checkups with patients during your clinical rotations or practice?*		
Yes	222	90.2
No	24	9.8
*Has your medical education influenced your attitude towards routine medical check‐ups?*		
Yes, positively	194	79.0
Yes, negatively	4	1.8
No influence	27	10.9
Not sure	21	8.3
*What factors influence your decision to schedule or not schedule a routine checkup?*		
Health concerns or symptoms	175	24.2
Lack of time	152	21.0
Lack of health insurance or cost concerns	98	13.6
No perceived need (feel healthy)	141	19.5
Forgetfulness	97	13.4
Availability of healthcare facilities	60	8.3
*What do you think are the main barriers to routine medical checkups?*		
Lack of time	116	15.8
Lack of awareness about the importance	200	27.2
Financial constraints or insurance issues	190	25.8
Fear of discovering health problems	118	16.0
Lack of healthcare providers in the area	43	5.8
Lack of trust in the healthcare system	69	9.4
*How can healthcare providers encourage the general population to undergo regular medical checkups?*		
Offering reminders or health campaigns	181	27.6
Providing convenient and flexible appointment times	169	25.7
Offering affordable or free checkups	115	17.5
Providing more information about the benefits of routine checkups	96	14.6
Reducing waiting times at healthcare facilities	96	14.6

Second, the research team created a set list of six items (Table 5) to quantify the barriers to checkups without using any existing qualitative research with this particular cohort or a validated measuring technique. Participants were unable to report significant obstacles that are well‐documented in South Asian medical student populations, such as embarrassment, a preference for self‐treatment, a sense of invincibility as a medical professional, or trouble navigating healthcare systems as a student. This restricts content validity and might have focused responses on pre‐established categories rather than accurately depicting the actual barrier landscape. Health surveys based on ad hoc item lists, as opposed to regularly developed and tested scales, run the danger of omitting important variables and generating inaccurate prevalence statistics for specific obstacles, as noted by Boateng et al. [3]. Prior to widespread implementation, future research should first identify the entire spectrum of barriers through qualitative interviews or focus groups with medical students. Based on the results, a barrier‐specific questionnaire should be developed and psychometrically validated.

Third, the methodology is purely descriptive and atheoretical, even if the study shows a correlation between greater knowledge scores and confidence in discussing checkups (*p* < 0.001, Table 6). No well‐known health behavior paradigm, such as the Theory of Planned Behavior, the Health Belief paradigm, or the Transtheoretical Model, was employed to organize the variables or provide an explanation for why personal action does not follow from solid knowledge. The study cannot determine which psychological factors motivate or inhibit students' preventative activity without evaluating notions like perceived susceptibility to disease, self‐efficacy, or cues to action. This significantly restricts the capacity to create focused, successful interventions. Felt susceptibility and felt advantages were the biggest behavioral predictors of routine health checkup attendance, according to Lian and colleagues' use of the Health Belief Model. These results could not have been obtained via a descriptive survey alone [4]. Future survey tools that incorporate a proven behavioral paradigm would enable researchers to go beyond simply characterizing the knowledge‐practice gap and instead understand and address it.

**Table 6:** Comparison of mean values of the knowledge of respondents towards routine medical check‐ups in relation to their attitude (*N* = 246).

	Mean (SD)	Test statistics	*p* value
*Do you personally schedule regular routine checkups even when you feel healthy?*			
Yes	3.58 (0.999)	−0.57	0.96
No	3.59 (0.967)		
*Have you ever recommended routine checkups to family or friends as a preventive health measure?*			
Yes	3.64 (0.947)	1.675	0.10
No	3.37 (1.062)		
*When was the last time you had a routine checkup?*			
Less than 6 months ago	3.73 (0.952)	1.281	0.28
6–12 months	3.61 (1.054)		
1–2 years ago	3.54 (0.962)		
More than 2 years ago	3.72 (0.886)		
Never	3.38 (0.973)		
*Do you feel confident in discussing the benefits of routine checkups with patients during your clinical rotations or practice?*			
Yes	3.67 (0.936)	4.177	< 0.001
No	2.84 (0.987)		
*Has your medical education influenced your attitude towards routine medical check‐ups?*			
Yes, positively	3.64 (0.935)	1.733	0.16
Yes, negatively	2.80 (1.483)		
No influence	3.52 (1.051)		
Not sure	3.35 (1.040)		

Fourth, the sample size of 246 was determined with a 5% margin of error in order to estimate a single population proportion: the prevalence of knowledge about checkups (Section 2.3). The independent *t*‐tests and one‐way analysis of variance used to compare knowledge scores across gender, age groups, and years of study did not undergo a separate power calculation (Table 4). With this sample size and no prior power estimate for these comparisons, the study may have been underpowered to detect small‐to‐moderate but clinically meaningful differences between groups. The finding that gender (*p* = 0.82), age (*p* = 0.93), and year of study (*p* = 0.55) were not significantly associated with knowledge could reflect a true null result—or it could be a false negative driven by insufficient sample size. Zhang and colleagues, studying health literacy in medical students in China with a substantially larger sample of 1524 participants, did detect significant differences by gender and academic year that smaller studies would have missed [5]. To guarantee that subgroup analyses are sufficiently powered and that negative results can be confidently interpreted, future research should do a priori power calculations independently for each planned comparison.

In conclusion, the lack of record‐based verification of self‐reported practices, the use of an unvalidated ad hoc barrier list, the absence of a theoretical behavioral framework, and the insufficient statistical power for subgroup comparisons collectively limit the precision and depth of the findings, even though Akram et al. make a significant contribution to the literature on preventive health behavior among medical students in Pakistan. In order to produce the solid, useful data required to create successful treatments that close the knowledge‐practice gap in this significant group, future research must address these methodological flaws.

## Author Contributions

**Muhammad Mehr Ali:** conceptualization, project administration, writing – original draft, writing – review and editing, validation. **Yousra Noor:** investigation, validation, writing – review and editing, writing – original draft. **Amna Khalid:** writing – original draft, writing – review and editing, investigation, validation.

## Funding

The authors have nothing to report.

## Ethics Statement

The authors declare that this manuscript has been prepared in accordance with Wiley's Best Practice Guidelines on Publishing Ethics and the principles of the Committee on Publication Ethics (COPE). The work has been conducted ethically and responsibly, without research misconduct including data fabrication, data falsification, plagiarism, duplicate publication, inappropriate authorship practices, or undeclared conflicts of interest. All authors have reviewed and approved the final manuscript and agree to be accountable for all aspects of the work.

## Data Availability

The data supporting the findings of this study are available from the corresponding author upon reasonable request. As this article is a Letter to the Editor and does not involve original participant‐level data collection, no additional datasets were generated or analyzed during the current study.
